# Operational research capacity building through the Structured Operational Research Training Initiative (SORT-IT) in China: implementation, outcomes and challenges

**DOI:** 10.1186/s40249-021-00865-w

**Published:** 2021-06-01

**Authors:** Ning Feng, Jeffrey Karl Edwards, Philip Odhiambo Owiti, Guo-Min Zhang, Zulma Vanessa Rueda Vallejo, Katrina Hann, Shui-Sen Zhou, Myo Minn Oo, Elizabeth Marie Geoffroy, Chao Ma, Tao Li, Jun Feng, Yi Zhang, Xiao-Ping Dong

**Affiliations:** 1 Center for Global Public Health, Chinese Center for Disease Control and Prevention, Beijing, China; 2 Department of Global Health, University of Washington, Seattle, WA USA; 3 The National Tuberculosis, Leprosy and Lung Disease Program, Nairobi, Kenya; 4 National Immunization Program, Chinese Center for Disease Control and Prevention, Beijing, China; 5 5Universidad Pontificia Bolivariana, Medellín, Colombia; 6 Sustainable Health Systems, Freetown, Sierra Leone; 7 Chinese Center for Disease Control and Prevention, National Institute of Parasitic Diseases, Shanghai, China; 8 Center for Operational Research, International Union Against Tuberculosis and Lung Disease, Mandalay, Myanmar; 9 Global AIDS Interfaith Alliance, San Rafael, CA USA; 10 National Center for Tuberculosis Control and Prevention, Chinese Center for Disease Control and Prevention, Beijing, China; 11 Chinese Center for Disease Control and Prevention, National Institute for Viral Disease Control and Prevention, Beijing, China

**Keywords:** Operational research, Capacity building, Disease control program

## Abstract

**Background:**

Chinese Center for Disease Control and Prevention (China CDC) introduced the Structured Operational Research Training Initiative (SORT IT) into China to build a special capacity and equip public health professionals with an effective tool to support developing countries in strengthening their operational research. The paper aims to investigate and analyze the implementation, outcomes and challenges of the first cycle of SORT IT in China.

**Main text:**

As a result of the successful implementation, SORT IT China, Cycle 1 has demonstrated fruitful outputs as exemplified by the 18-month follow-up to the post-training initiatives of the twelve participants, who all achieved the four milestones required by SORT IT. Eleven of twelve (92%) manuscripts generated that focused on the prevention and control of malaria, influenza, HIV/AIDS, hepatitis B, schistosomiasis, tuberculosis and Japanese encephalitis were published by peer-reviewed international journals with the impact factor ranging from 2.6 to 4.8. The most up-to-date citation count on February 19, 2021 was 53 times out of which 31 times were cited by Science Citation Index papers with 94.827 impact factor in total. Six senior professionals from China CDC also facilitated the whole SORT IT training scheme as co-mentors under the guidance of SORT IT mentors. The twelve participants who gained familiarity with the SORT IT courses and training principles are likely become potential mentors for future SORT IT, but they as the non-first language speakers/users of English also faced the challenge in thoroughly understanding the modules delivered in English and writing English academically to draft the manuscripts.

**Conclusion:**

The outcomes from the first cycle of SORT IT in China have led to studies contributing to narrowing the knowledge gap among numerous public health challenges nationally and internationally. It is believed the researchers who participated will continue to apply the skills learned within their domain and help build the training capacity for future operational research courses both in China and in developing countries with similar needs.

**Graphic Abstract:**

**Supplementary Information:**

The online version contains supplementary material available at 10.1186/s40249-021-00865-w.

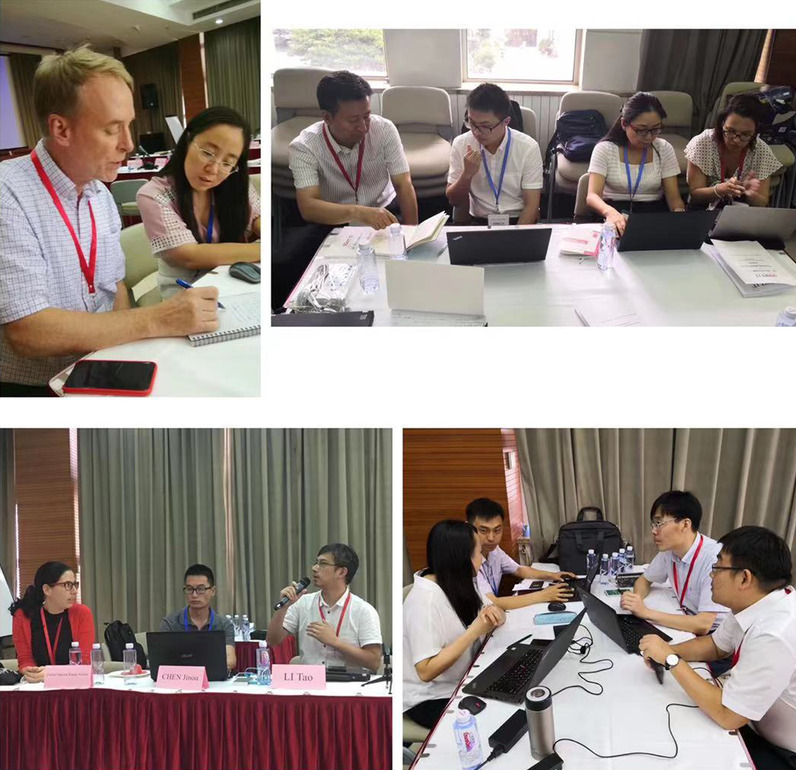

## Background

As the global community has continued to grapple with health threats, the global partnerships should share increasingly greater and more important responsibilities to respond to the global health threats through cross-sectoral, inter-governmental and interdisciplinary collaboration. Being as a national professional institution specialized in diseases control and prevention in China with its services covering 1.4 billion population, Chinese Center for Disease Control and Prevention (China CDC) does not only boast internationally or domestically reputable scientists/experts, but also has a comprehensive diseases control and prevention networks staffed with experienced professional teams throughout the country, therefore bringing an unshakable obligation for China CDC to rise and respond to the threats that jeopardize the lives and wellbeing of the public.

China CDC is actively participating in global public health cooperation including China-Africa public health cooperation. In this connection, China CDC has launched a special program to enhance the capacity in global public health development cooperation that is supported financially mainly by resources from Ministry of Finance of People’s Republic of China and jointly by multiple international and domestic funding sources. The program aims to successively organize and implement a series of capacity-building sessions attended by both Chinese and foreign professionals in public health and delivered both in China by high profile international facilitators and in other countries by a team of Chinese experts. Participants are expected to develop enhanced and comprehensive capacities covering various links of global health cooperation, such as having adequate theoretic knowledge of disease control and prevention, developing a communication mechanism and working platform, establishing a talent pool, providing on-site technical support and conducting international emergency response capacity building, among others. Specifically, Chinese public health professionals need to build a special capacity and be equipped with an effective tool to support developing countries to strengthen their operational research (OR) within public health systems. To respond to such an urgent need, China CDC thus has introduced the Structured Operational Research Training Initiative (SORT IT) into China.

SORT IT is a validated, modular training program that concentrates on capacitating OR in public health to respond to critical programmatic challenges that can significantly impact healthcare practice and policy. SORT IT was developed collaboratively in 2009 by the International Union Against Tuberculosis and Lung Disease and Médecins Sans Frontières Brussels-Luxembourg (MSF). In 2013, SORT IT was endorsed by the WHO-based Special Programme for Research and Training in Tropical Diseases (WHO/TDR).

The principle goals of a SORT IT training cycle containing three modules is to help hosting countries to develop adequate and sustainable operational research (OR) capacity in public health programs embodying the countries’ priorities and lead to improved program performance. SORT IT has four milestones with strict deadlines required for participants to advance to the next module, with the final step leading to achieving a training certificate and submission of a manuscript to an open-access peer-reviewed journal. Since 2009, more than 80 courses have been held globally and have led to significant impact on the shaping of national and international public health policy and practice.

The mission of the 1^st^ Cycle of SORT IT in China was not only to achieve the general goals of SORT IT itself, but also to foster Chinese facilitators who can skillfully utilize SORT IT in supporting public health professionals of developing countries. We hereby present a summary of the first SORT IT courses held within China that was sponsored by the China CDC, MSF and WHO/TDR. During 2018, two SORT IT workshops in China were held, Modules 1 and 2 delivered in July and Module 3 in December. Six participants from China CDC and six from provincial CDCs received hands-on training by six international mentors and six co-mentors from the China CDC, focusing on both emerging and remerging communicable diseases.

## Main text

### The output of SORT IT China, Cycle 1

SORT IT China, Cycle 1 has demonstrated fruitful output as exemplified by the 18-month follow-up to the post-training initiatives of the twelve participants, who all achieved the four milestones required by the project. During this period following the training, eleven of twelve (92%) manuscripts generated were published by peer-reviewed international journals, with the impact factor of the publishing journals ranging from 2.6 to 4.8. As of 19 February 2021, the citation count was 53 times in which 31 times were cited by papers published in journals indexed in Science Citation Index (SCI). The research findings presented in these papers included the prevention and control of malaria, influenza, HIV/AIDS, hepatitis B, schistosomiasis, tuberculosis and Japanese encephalitis, with the details shown in Additional file 1: Table S1.

Six senior professionals from China CDC facilitated the whole SORT IT training scheme as co-mentors under the guidance of SORT IT mentors. Meanwhile, the twelve participants gained familiarity with SORT IT courses and training principles so as to enable to become potential mentors for future SORT IT.

### Successes and challenges of SORT IT China, Cycle 1

Following the conclusion of each module, an anonymous survey was completed by the participants and a roundtable discussion was held with all facilitators and participants regarding the success and challenges encountered. The primary achievements in this SORT IT training reported included: (1) successful training of 12 Chinese public health professionals in conduction OR which led to submission of scientific manuscripts; (2) Mentoring of 12 Chinese CDC leadership scientists, who will hopefully go on to conduct further SORT-IT courses in China and developing countries; (3) improving collaboration between China CDC, MSF and WHO.

The challenges reported included: (1) receiving training in the participants’ non-native language; (2) the limited time of each module; (3) technical writing in the participants’ non-native language.

Despite these challenges, the SORT IT workshop achieved all targeted goals, which has reflected the commitment and work of those involved in the preparation before the course started, the logistics involved during each module and the follow up provided to the participants following completion. At this time, the China CDC is looking forward to starting its next SORT IT training in China and hoping to expand teaching capacity to developing country partners in the future.

### Benefits provided by the feedbacks of the participants

Through the Focus Group Discussion, all participants have provided positive feedbacks on the enormous benefits they have reaped from the SORT IT workshop. They considered that the training had widened their global vision and horizon, enhanced their capacity to draft research manuscripts in English which in turn will advance their own professional performance, and enhanced the capacity of OR among the public health professionals in China.

The manuscripts generated by the SORT IT project have played a positive role in the control of the following diseases such as Tuberculosis, HIV, malaria, flu and schistosomiasis. One manuscript provided evidence to the revision of the Working Guidelines for the National Program for Prevention of Mother-to-Child, and another one informed the development of China’s control and prevention strategy for imported malaria in the context of malaria elimination. There was also a manuscript that have been directly cited by the *Recommendation Report on the Flu Vaccine Immunization Strategy through Fall and Winter,* and *Report to Recommend the Priority Groups to be Inoculated with the Flu Vaccine* t*hrough Fall and Winter*. The two documents have provided key technical support for National Health Commission of the People’s Republic of China to develop the flu control technical guidelines, bring the outbreak of respiratory diseases under control and minimize the damages from the outbreak to the health of the Chinese population.

## Conclusions

The outcomes thus far from the first cycle of SORT IT in China have led to studies contributing to narrowing the knowledge gap among numerous public health challenges nationally and internationally. It is believed the researchers who participated will continue to apply the skills learned within their domain and help build the training capacity for future operational research courses both in China and in developing countries with similar needs.

## Supplementary Information

**Additional file 1: Table S1. **Titles of the manuscripts, names of peer-reviewed scientific journals, SCI, Access and Citation.

## Data Availability

All data generated or analyzed during this comment are included in this published article and its supplementary information files.

## Abbreviations

China CDC: Chinese Center for Disease Control and Prevention
SORT IT: Structured Operational Research Training Initiative
OR: Operational Research
SCI: Science Citation Index
WHO/TDR: World Health Organization-based Special Programme for Research and Training in Tropical Diseases
MSF: Médecins Sans Frontières
JIF: Journal impact factor
