# Supplementary material for: Operational research capacity building through the Structured Operational Research Training Initiative (SORT-IT) in China: implementation, outcomes and challenges
Source: Infect Dis Poverty. 2021 Jun 1;10:80. doi: 10.1186/s40249-021-00865-w (PMC8170796; doi:10.1186/s40249-021-00865-w)
Supplement: Supplementary file 1 — Additional file 1: Table S1. Titles of the manuscripts, names of peer-reviewed scientific journals, SCI, Access and Citation. [file 40249_2021_865_MOESM1_ESM.docx]

**4. Supplementary file**

The publishing and citation status of the manuscripts generated in SORT IT China, Cycle 1 are shown in table 1.

**Table 1. Titles of the manuscripts, names of peer-reviewed scientific journals, SCI, Access and Citation**

| **Code** | **Title of Manuscript** |  | **Name of Journal** | **SCI** | **Access** | **Citation (times, SCI number, SCI total)** |
| --- | --- | --- | --- | --- | --- | --- |
| 1 | Change patterns of oncomelanid snail burden in areas within the Yangtze River drainage after the three gorges dam operated^[1]^ |  | *BMC Infectious Diseases of Poverty* | *3.123* | *911* | *9, 8, 24.620* |
| 2 | Monitoring and evaluation of intervals from onset of fever to diagnosis before “1-3-7” approach in malaria elimination: a retrospective study in Shanxi Province, China from 2013-2018^[2]^ |  | *BMC Malaria Journal* | *2.798* | *1010* | *8, 1, 2.631* |
| 3 | Outcomes of the national programme on prevention of mother-to-child transmission of hepatitis B virus in China, 2016-2017^[3]^ |  | *BMC Infectious Diseases of Poverty* | *3.123* | *1000* | *7, 4, 6.482* |
| 4 | Compliance among infants exposed to hepatitis B virus in a post-vaccination serological testing program in four provinces in China^[4]^ |  | *BMC Infectious Diseases of Poverty* | *3.123* | *1202* | *1, 1, 3.297* |
| 5 | Role of community-based active case finding in screening tuberculosis in Yunnan province of China^[5]^ |  | *BMC Infectious Disease of Poverty* | *3.123* | *3199* | *9, 3, 8.084* |
| 6 | Knowledge, attitudes, and behaviors of influenza vaccination in China: a cross-sectional study in 2017/2018^[6]^ |  | *Vaccines* | *4.760* | *NA* | *5, 4, 11.669* |
| 7 | Reduction patterns of Japanese encephalitis incidence into long-term expanded program on immunization in Yunnan Province, China^[7]^ |  | *BMC Infectious Diseases of Poverty* | *3.123* | *900* | *2, 2, 13.272* |
| 8 | Effect of the Xpert MTB/RIF on the detection of pulmonary tuberculosis cases and rifampincin resistance in Shanghai, China^[8]^ |  | *BMC Infectious Diseases* | *2.565* | *1001* | *2, 1, 2.268* |
| 9 | HIV testing uptake and yield among sexual partners of HIV-positive men who have sex with men in Zhejiang Province, China, 2014- 2016: A cross-sectional pilot study of a choice- based partner tracing and testing package^[9]^ |  | *PLOS ONE* | *2.875* | *NA* | *1, 1, 5.553* |
| 10 | Imported malaria cases in former endemic and non-malaria endemic areas in China: are there differences in case profile and time to response?^[10]^ |  | *BMC Infectious Diseases of Poverty* | *3.123* | *1374* | *9, 6, 16.951* |
| 11 | Prevalence of tuberculosis among close contacts of index cases in 27 universities in Beijing, China, 2017-18^[11]^ |  | *Biomedical and Environmental Sciences* | *2.626* | *NA* | *0, 0, 0* |
